# Nest initiation and flooding in response to season and semi-lunar spring tides in a ground-nesting shorebird

**DOI:** 10.1186/s12983-019-0313-1

**Published:** 2019-05-23

**Authors:** Silvia Plaschke, Martin Bulla, Medardo Cruz-López, Salvador Gómez del Ángel, Clemens Küpper

**Affiliations:** 1 Institute for Biology, University of Graz, Universitätsplatz 2, 8010 Graz, Austria; 2 Max Planck Institute for Ornithology, Eberhard-Gwinner-Str., 82319 Seewiesen, Germany; 3 NIOZ Royal Netherlands Institute for Sea Research, Department of Coastal Systems and Utrecht University, P.O. Box 59, 1790 AB Den Burg, The Netherlands; 4 Department of Ecology, Faculty of Environmental Sciences, Czech University of Life Sciences Prague, 165 21 Prague, Czech Republic; 5 Posgrado en Ciencias del Mar y Limnología, Universidad Nacional Autónoma de México, Ciudad Universitaria, 04510 Cd. México, Mexico

**Keywords:** *Charadrius nivosus*, Nest flooding, Ground-nesting shorebirds, Nest initiation schedule, Semi-lunar cycle, Snowy plover, Spring tide rhythm

## Abstract

**Background:**

Marine and intertidal organisms face the rhythmic environmental changes induced by tides. The large amplitude of spring tides that occur around full and new moon may threaten nests of ground-nesting birds. These birds face a trade-off between ensuring nest safety from tidal flooding and nesting near the waterline to provide their newly hatched offspring with suitable foraging opportunities. The semi-lunar periodicity of spring tides may enable birds to schedule nest initiation adaptively, for example, by initiating nests around tidal peaks when the water line reaches the farthest into the intertidal habitat. We examined the impact of semi-lunar tidal changes on the phenology of nest flooding and nest initiation in Snowy Plovers (*Charadrius nivosus*) breeding at Bahía de Ceuta, a coastal wetland in Northwest Mexico.

**Results:**

Using nest initiations and fates of 752 nests monitored over ten years we found that the laying season coincides with the lowest spring tides of the year and only 6% of all nests were flooded by tides. Tidal nest flooding varied substantially over time. First, flooding was the primary cause of nest failures in two of the ten seasons indicating high between-season stochasticity. Second, nests were flooded almost exclusively during the second half of the laying season. Third, nest flooding was associated with the semi-lunar spring tide cycle as nests initiated around spring tide had a lower risk of being flooded than nests initiated at other times. Following the spring tide rhythm, plovers appeared to adapt to this risk of flooding with nest initiation rates highest around spring tides and lowest around neap tides.

**Conclusions:**

Snowy Plovers appear generally well adapted to the risk of nest flooding by spring tides. Our results are in line with other studies showing that intertidal organisms have evolved adaptive responses to predictable rhythmic tidal changes but these adaptations do not prevent occasional catastrophic losses caused by stochastic events.

## Introduction

All living organisms, from the simplest prokaryote to the most complex eukaryote, face the rhythmic changes of the Earth’s periodic environment. The tidal, daily, seasonal, as well as multiyear environmental periodicities are almost exclusively induced by the movements of the Earth relative to the Sun and the movements of the Moon relative to the Earth [1]. Organisms that align their physiology and behaviour to these persistent environmental cycles can enhance their survival and reproductive success [2–5].

**Fig. 1:**
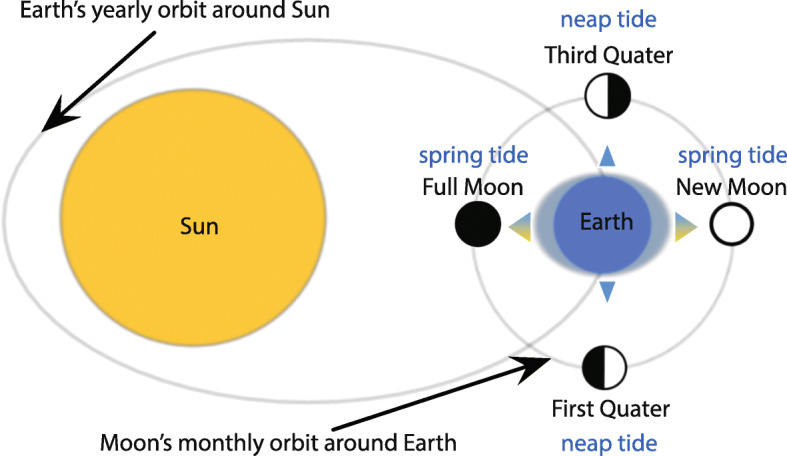
Variation in tide heights during a lunar month. Spring tides occur shortly after new and full moon, twice during a lunar month. During spring tides the Sun and Moon are aligned and their combined gravitational forces lead to the largest difference between high and low tide resulting in particularly high water lines at high tides. In contrast, neap tides occur shortly after the first and third quarter of the Moon. During neap tides, the gravitational forces are at minimum leading to the smallest difference between high and low tide

Moon-driven environmental cycles such as lunar illumination and raising and falling of sea water levels (tides) vary in their periodicity (Fig. 1). Lunar illumination changes over 24.8 h (the lunar day with the Moon rotating once around the Earth) and over 29.5 days (the lunar month when the Moon cycles once through its phases). Correspondingly, sea levels rise and fall every ~ 12.4 h (tidal cycle), maximum and minimum high tides (i.e. spring and neap tides, respectively) occur once during every semi-lunar cycle (~ 14.75 days) around new and full moon (spring tides) or around half moon, i.e. the Moon’s cycle’s 1st and 3rd quarter (neap tides). Consequently, a multitude of biological functions, including oxygen consumption, foraging activity, or reproduction, follow tidal and/or lunar cycles in a broad array of organisms [1, 6–13].

The organisms breeding in intertidal wetlands are subject to predictable flooding caused by high tides. Organisms shall be well adapted to such rhythmic daily and semi-lunar flooding. However, tide heights are also modulated by weather conditions. For example, onshore winds will push the waterline further inland. Occasionally, this may lead to extreme and unpredictable flooding events, especially during periods of rough weather [10, 14–20]. How do organisms adapt to the risk of nest flooding? There are two main mechanisms, spatial and temporal adaptation. First, for spatial adaptation, individuals may choose sites that are perceived as safe from flooding for both them and their offspring. For example, some ground-nesting birds construct elevated nest mounts or select elevated nest sites that won’t be reached by spring tides during the breeding season [10, 15, 17, 18, 20]. Such elevated nests, however, may make nests more conspicuous to predators [17, 20, 21]. Alternatively, parents may select nest sites far away from the highest tide line. This may reduce the risk of flooding but increases the distance to favourable foraging sites for parents and offspring [22]. In addition, sites far away from the highest tide line tend to have higher vegetation, which may hinder early visual detection of predators [23].

Second, for temporal adaptation, tidal organisms may time their reproduction to periods when conditions are favourable for their offspring [12, 24]. For example, for terrestrial ground-nesting organisms initiating nests right after spring tides will typically reduce nest vulnerability to flooding since these nests may be exposed to fewer spring tides and the nests are placed above the current highest tide line. In contrast, parents that initiate nests during neap tides will lack information about the highest tide line and hence their nests should be more vulnerable to spring tide flooding.

Whereas spatial adaptation in relation to tidal flooding is reasonably well studied, temporal adaptation of nesting has received less attention [10, 25], and the available studies lack statistical models that take the circular properties of tidal rhythms into account (but see [20]).

Here, we investigate temporal dynamics of nest flooding and nest initiation in relation to spring tide cycle in the Snowy Plover (*Charadrius nivosus*). This ground-nesting shorebird breeds in open sparsely vegetated habitat at Pacific and Gulf coasts and interior salt flats of North and South America [26, 27] where it frequently experiences tidal flooding and/or rough weather events [14, 28–32]. The nesting period, i.e. the period between first egg laid and last chick hatched, varies but typically lasts about 32 days ([27]; CK, MC-L, SGA unpublished data) meaning that nests are typically exposed to two spring tides. Incubation is shared by male and female but shows substantial geographic variation in length and parental cooperation [27, 33–35]. The precocial chicks leave the nest scrape within a few hours after hatching and the family then moves to a brood territory typically at the shore often several kilometers away from the nest.

Specifically, we collected data on nest initiation and fates of 752 Snowy Plover nests over ten years at Bahía de Ceuta, a tidal wetland in Northwest Mexico. At this site, the laying season of Snowy Plovers, here defined as the period between first and last initiated clutch in a given year, starts when the breeding grounds are laid bare by receding water and ends when rising tidal amplitude and rain flood the breeding grounds [14, 36]. Throughout the laying season tide height is increasing. However, increasing seasonal temperatures lead to high evaporation and a net loss of surface water from April until the start of the rain season. As a consequence, the water body and with it the nesting plovers recede towards areas in the northwest that still provide access to water but these areas are more susceptible for tidal flooding (CK, MCL, SGA personal observations.

**Fig. 2:**
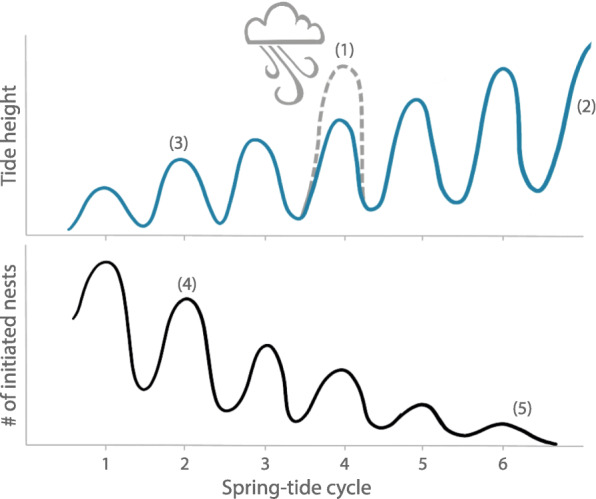
Schematic representation of predictions for nesting phenology of Snowy Plovers at Bahía de Ceuta, Mexico. Numbers (1–5) indicate each prediction. Stochastic extreme weather events that occur in some years but not in others lead to unpredictable high spring tides (dotted grey line) causing between-year variation in nest flooding (1). Rising daily tide heights (undulating blue line) over the course of the laying season increase flooding of nests laid later in the season (2). Nests initiated around spring tide have a lower flooding risk than those initiated around neap tide because parents can place the nest in a safe distance from the highest water line (3). Semi-lunar variation in nest flooding will lead to adaptive nest initiation patterns that follow the semi-lunar spring tide cycle with more nests initiated around spring tide and less around neap tide (4). The semi-lunar periodicity in nest initiation will weaken as the season draws to its end since parents are pressed for time as the breeding site will be flooded at the end of the breeding season (5)

To examine the relationship between the semi-lunar cycle, nest flooding and initiation in this population we tested the following five predictions (Fig. 2). (1) Between-year nest flooding will be stochastic because tidal amplitude varies with weather conditions, and rough weather hits coastal areas stochastically over years [19, 37]. (2) Late nests will be more likely flooded than early nests because the tidal amplitudes increase throughout the laying season and because rough weather, and hence unusually high tides, are more common at the end of the season at this site (CK, MCL, SGA personal observations). (3) Nests initiated around spring tide will be less likely flooded than nests initiated around neap tide. This is because parents experience how far the water line advances around spring tides and can choose their nest location accordingly. (4) If flooding risk varies throughout the spring tide cycle, adaptive nest initiation will show a similar periodicity as the semi-lunar spring tide cycle with nest initiation peaking around spring tides and reduced nest initiation around neap tides. (5) This semi-lunar periodicity in nest initiation will be stronger early and weaker late in the season as early plovers can optimally time their nest initiation whereas late plovers are more rushed to complete the nesting before the end of the laying season as the nesting areas will get completely flooded shortly after the start of the rain season.

## Methods

### Data collection

We monitored a population of Snowy Plovers breeding at Bahía de Ceuta, a tidal wetland in Northwest Mexico (23.9° N, 106.95° W), between 2006 and 2016, with exception of 2014. From April to July 70 to 200 plovers breed on the salt flats. We searched for nests by identifying incubating or nest-building parents with binoculars and/or spotting scopes from a car or mobile hide [38]. Upon finding, we recorded nest position with a handheld GPS, noted clutch size and floated the eggs in lukewarm water to determine their age based on the stage of the youngest egg [38]. This method determines the onset of incubation up to a clutch age of ten days. We trapped adults on nests using funnel traps and marked them with a unique combination of metal and colour rings (e.g. [14, 39]. We determined the sex of parents based on plumage differences and differences in capture time [27, 34]. For all birds captured between 2006 and 2012, the phenotypic sexing was confirmed by molecular analysis (details given in [34]).

We visited nests every three to five days until incubation day 20 and at least once every 48 h thereafter to determine nest fate, i.e. hatched, depredated, flooded, abandoned, or unknown. We further differentiated nests presumably flooded by tides, i.e. nests that failed because they were covered by surface water (hereafter ‘flooded’) from nests drowned by rainfall, i.e. nests found flooded just after heavy rainfall.

We defined ‘nest initiation’ as the day when the first egg was laid whereas the ‘onset of incubation’ as the day when the last egg was laid. We assumed a 5 day period for females to complete a three-egg clutch and a 3 day period to complete a two-egg clutch [27]. We further assume a 25-day long incubation period. This is based on 51 hatched nests that we found during laying (median [range] = 25 [23 to 29] days) and matches the incubation period assumed in earlier studies [36, 40]. Thus, if a nest was found during egg laying (*N* = 93) or was found with a complete clutch that was 10 days old or younger (*N* = 616), we estimated its nest initiation based on the clutch size and the age of the youngest egg. One three-egg clutch was found with one egg but lacked floating information and we assumed that the egg was laid the day before finding it. We calculated nest initiation of 25 clutches found with an incubation age of 11 days or older and of further 18 hatched clutches based on their hatching dates. Thirteen failed nests found with an incubation age of 11 days or older were excluded from the analysis because we could not determine their nest initiation. This resulted in 746 nests with records for all variables of interest (i.e. nest coordinates, clutch size, nest initiation and fate, sex and identity of the parents). For further six nests with missing nest locations we imputed their coordinates using the median latitude and median longitude of the nests laid ± 5 days around the nest initiation date of the given nest as imputation rather than an exclusion of cases with missing data increases the precision of the analysis [41]. The final clutch size was three eggs in 652 nests (86.7%), two in 78 nests (10.4%) and one egg in 22 nests (2.9%).

**Fig. 3:**
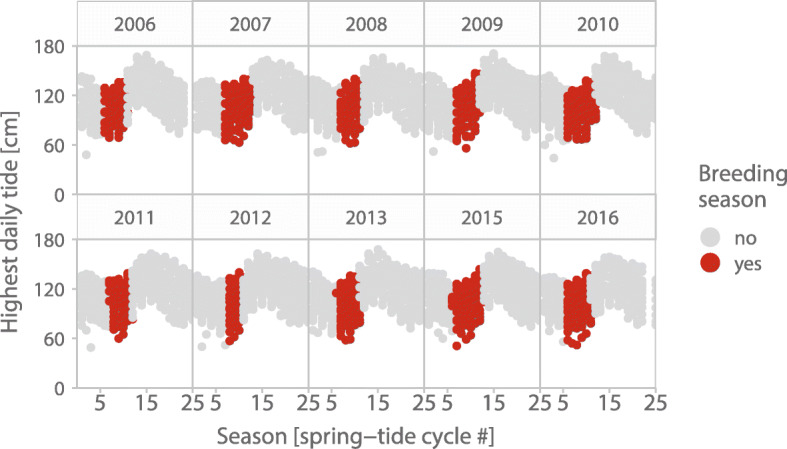
Between- and within-year variation in daily tide height at Bahía de Ceuta, Mexico. Each point depicts the predicted highest daily tide, its color indicates laying season (red) or non-laying season (grey). For yearly variation of high tides across the spring tide cycle

We downloaded the daily tide height predictions from (ref. [42]) for the port of Mazatlán (N 23.25°, W 106.42°), the nearest location to Bahía de Ceuta with available tide height data approximately 95 km south of our study site. We used the highest tide height for any given day (Fig. 3). For 10 days the highest tide height was missing and we imputed the missing values as the mean of highest tide heights from previous and next days. The new and full moon data for Mazatlán were downloaded from timeanddate.com and used to define the dates of spring tides.

### Statistical analysis

#### General procedures

We used R, version 3.5.1 [43] for statistical analyses and the ‘lme4’ R-package [44] for fitting mixed-effect models with restricted maximum likelihood. We used the ‘sim’ function from the ‘arm’ R package and non-informative prior-distribution [45, 46] to create a sample of 5000 simulated values for each model parameter (i.e. posterior distribution). We report effect sizes and model predictions by the medians, and the uncertainty of the estimates and predictions by the Bayesian 95% credible intervals represented by 2.5 and 97.5 percentiles (95% CI) from the posterior distribution of the 5000 simulated or predicted values [47]. We estimated the variance components with the ‘lmer’ or ‘glmer’ function from the ‘lme4’ R package [44] and report those as the percentage of explained random variance. We used three sets of models to investigate our five predictions. ‘Day of spring tide cycle‘ indicates time since the last spring tide with day one representing the day of a spring tide. To overcome the circular properties of time whenever we investigated effect of time within spring tide cycle, we transformed ‘day of spring tide cycle’ to radians (2 * π * ‘days of spring tide cycle’ / length of the given spring tide cycle [~ 14.75 days]; note that spring tide cycles vary in length, in our data by 1.6 days). We then fitted the radians to sine and cosine [35]. When investigating the seasonal effect, we defined the progress of the season by 'spring tide cycle number'; the first spring tide cycle in a given year corresponds to the cycle when the first nest was initiated. To control for non-independence of data points, we also specified year, spring tide cycle number and female identity as random intercepts.

We fitted all models with two different error structures: Gaussian and Non-Gaussian errors. Despite violating some model assumptions, Gaussian models perform well on large datasets where the response variable follows binomial or Poisson distribution [48]. Consistent with this, we observed that the Gaussian models fitted the data better than the binomial or Poisson models and used Gaussian models for the graphic representation of our results but present both models with Gaussian and Non-Gaussian errors in the Results (Tables 1 and 2).

**Table 1:** Temporal variation in nest flooding

		Response – flooded (0 = no, 1 = yes)	Gaussian			Binomial		
Model	Effect type	Effect	Estimate	95% CI		Estimate	95% CI	
Complex	Fixed	Intercept	0.155	−0.005	0.317	−9.3	−13.4	−5.2
		Nest latitude	−0.078	−0.099	−0.055	−2.8	−4.4	−1.2
		Spring tide cycle number	0.164	0.052	0.275	4.9	3	6.9
		Cos (Day of spring tide cycle)	−0.01	−0.038	0.017	−0.7	−1.8	0.4
		Sin (Day of spring tide cycle)	−0.03	−0.058	−0.002	−1.2	−2.2	−0.1
	Random (variance)	Year (intercept)	17%			100%		
		Spring tide cycle number (intercept)	33%			0%		
		Female ID (intercept)	1%					
		Residual	49%					

The posterior estimates (medians) of the effect sizes with the 95% CIs derived from a posterior distribution of 5000 simulated values generated by the ‘sim’ function in R. Variance components were estimated by the ‘lmer’ function in R. To account for non-independence of data points, ‘Female ID’, ‘Year’ and ‘Spring tide cycle number’ (i.e. time within the laying season) were fitted as random intercepts. ‘Spring tide cycle number’ is standardized within the year so that the first spring tide cycle in the given year corresponds to the cycle when the first nest was initiated. This variable, as well as ‘Nest latitude’, were z-transformed (mean-centred and divided by standard deviation). ‘Day of spring tide cycle’ was transformed to radians (2 * number of days after the last spring tide * π/length of the given spring tide cycle [~ 14.75]) and fitted as sine and cosine of radians. Note that despite violating some model assumptions our Gaussian model fits the data better and, unlike our binomial model, also accounts for spatial auto-correlation in residuals. The binomial model lacks female identity as random intercept because models with female identity did not converge

**Table 2:** Temporal variation in nest initiation

		Response - # of initiated nests	Gaussian			Poisson		
Model	Effect type	Effect	Estimate	95% CI		Estimate	95% CI	
Complex	Fixed	Intercept	0.943	0.64	1.247	−0.302	−0.591	−0.02
		Spring tide cycle number	−0.195	−0.325	−0.065	−0.236	−0.375	−0.105
		Cos (Day of spring tide cycle)	0.105	0.002	0.208	0.096	−0.016	0.202
		Sin (Day of spring tide cycle)	0.043	−0.09	0.174	0.038	−0.103	0.176
		Cos × Spring tide cycle number	−0.06	−0.163	0.041	−0.048	−0.168	0.069
		Sin × Spring tide cycle number	0.016	−0.122	0.147	0.019	−0.127	0.168
	Random (variance)	First or second half : Spring tide cycle : Year (intercept)	9%			29%		
		Spring tide cycle : Year (intercept)	7%			19%		
		Year (intercept)	13%			40%		
		Residual – Gaussian/Observation (intercept) - Poisson	71%			12%		
Simple	Fixed	Intercept	1.349	0.954	1.747	0.205	−0.193	0.575
		Spring tide cycle number	−0.11	−0.188	−0.034	− 0.138	−0.216	−0.059
		Cos (Day of spring tide cycle)	0.104	0.001	0.211	0.033	−0.114	0.171
		Sin (Day of spring tide cycle)	0.04	−0.098	0.17	0.108	0.002	0.214
	Random (variance)	First or second half : Spring tide cycle: Year (intercept)	9%			30%		
		Spring tide cycle: Year (intercept)	7%			19%		
		Year (intercept)	13%			40%		
		Residual – Gaussian/Observation (intercept) - Poisson	72%			12%		

The posterior estimates (medians) of the effect sizes with the 95% CIs derived from a posterior distribution of 5000 simulated values generated by the ‘sim’ function in R. Variance components were estimated by the ‘lmer’ function in R. To account for non-independence of data points ‘Year’, ‘Spring tide cycle number’ within year and indication whether the nest was initiated in the ‘First or Second half’ of the spring tide cycle were fitted as random intercepts. Overdispersion was modelled by adding ‘Observation’ level as random intercept. ‘Spring tide cycle number’ is standardized within the year, so that the first spring tide cycle in the given year corresponds to the cycle when the first nest was initiated. ‘Day of spring tide cycle’ was transformed to radians (2 * number of days after the last spring tide * π/length of the given spring tide cycle [~ 14.75]) and fitted as sine and cosine of radians

#### Temporal variation in nest flooding

In the first set of models (Table 1), we investigated predictions 1 to 3 that are related to nest flooding. We used only flooded, hatched and unhatched nests (*N* = 476 nests) and specified whether the nest was flooded (1) or not (0). We fitted ‘Year’ as a random effect to the models to investigate prediction 1. We investigated whether flooding was related to the nest initiation date within the season (prediction 2) and within the spring tide cycle (prediction 3). We controlled for the latitudinal changes in nest initiation by entering nest latitude as a fixed effect. We then fitted two models, in one we specified the errors of the response as Gaussian, in the other as binomial.

#### Temporal variation in nest initiation

In the second set of models (Table 2), we used all nests (*N* = 752). We specified the number of initiated nests at any given day within a laying season as response variable and investigated how the response changed over the semi-lunar cycle (day of spring tide cycle, prediction 4) and with season (spring tide cycle number). In addition to the original random structure, we also fitted part of spring tide cycle (first or second) as random intercept. Again, we fitted two models, in one we specified the errors of the response as Gaussian, in the other as Poisson. In the Poisson model we controlled for over-dispersion by adding observation level as random intercept. To test prediction 5, we fitted the very same two models, but with interaction between ‘spring tide cycle number’ and ‘day of spring tide cycle’ (Table 2, complex models). To ensure that nest initiation was indeed driven by predicted changes of highest daily tides, we ran a third set of models. These models mirrored the second set, except that we used the ‘highest daily tide height’ (in cm) instead of ‘day of spring tide cycle’ as a predictor.

## Results

### Temporal variation in nest flooding

**Fig. 4:**
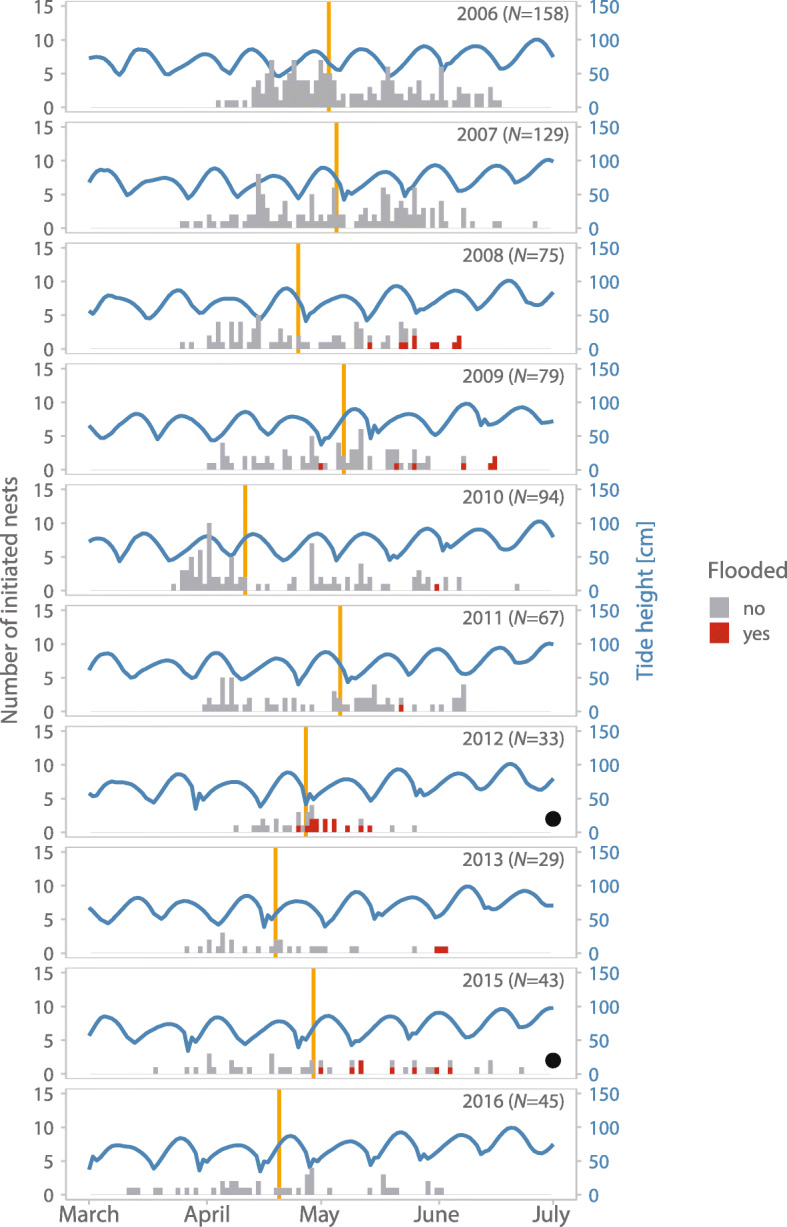
Number and fate of Snowy Plover nests initiated across years, laying season and spring tide cycle. Each bar represents one day, the color of the bar indicates fate of the nest (red – flooded by tides, *N* = 43; grey – not flooded including nests with unknown fate, *N* = 709). The undulating blue line represents the predicted highest daily tide and each hump indicates one spring tide cycle. The vertical orange line marks the median of the nest initiation date for each year. The black dots at the bottom-right mark the years when flooding was the primary cause of nest failure

**Fig. 5:**
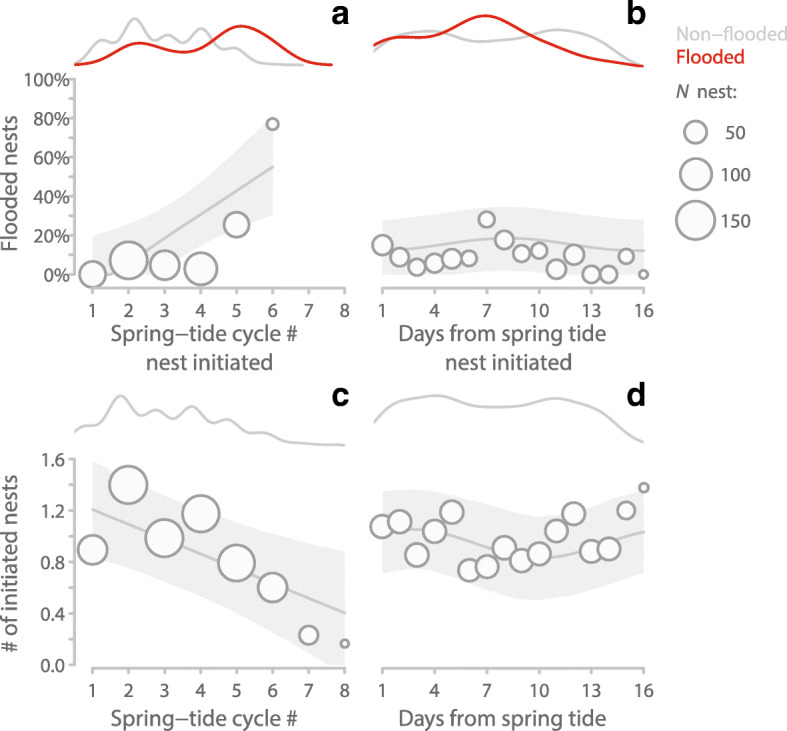
Nest flooding and initiation in relation to season and spring tide cycle. **a-b**, Percentage of flooded Snowy Plover nests in relation to time of the laying season, as indicated by spring tide cycle # (**a**), and in relation to day of spring tide cycle (**b)** when parents initiated their nest. Circles represent percentage of flooded nests initiated within each spring tide cycle (**a**) and within each day of the spring tide cycle (**b**). The lines at the top of the panels indicate Kernel-densities for non-flooded nests (grey) and flooded nests (red). *N* = 476 hatched, unhatched or flooded nests initiated by 266 females over ten years and six spring tide cycles. **c-d,** Number of initiated nests in relation to time of the laying season (**c**), and day within the spring tide cycle (**d**). Circles represent mean number of nests initiated for each spring tide cycle (**c**) or each day of the spring tide cycle (**d**). The lines at the top of the panels indicate Kernel-densities. *N* = 776 days from ten laying seasons encompassing 62 semi-lunar spring tide cycles. **a**-**d**, Circle size indicates the number of initiated nests in the particular spring tide cycle (**a**, **c**) or day (**b**, **d**). The lines and the shaded areas represent model predictions with 95% CI based on posterior distributions of 5000 simulated values generated from ‘simple Gaussian model’ outputs (Tables 1 and 2) using the ‘sim’ function in R [45] while keeping the other predictors constant. Note that in (**a**, **b**) the 7th semi-lunar cycle contained only two data-points and hence these were modeled as the 6th cycle

Of 752 initiated nests 413 hatched (55%), 20 never hatched (3%) and only 43 nests (6%) were flooded by tides. Consistent with our first prediction, years differed greatly in the number of flooded nests (range: 0 to 13 nests, i.e. 0 to 39% of initiated nests) as well as in the number of initiated nests (range: 29 to 158 nests) and flooding was the primary reason for nest failure in two of the ten seasons (2012 and 2015; Fig. 4). Indeed, ‘year’ explained a substantial amount of phenotypic variance (Tables 1, and 2). Consistent with our second prediction, nests that were initiated later in the laying season were more likely to get flooded than earlier nests (Figs. 4 and 5a; Table 1). Finally, consistent with our third prediction, nests initiated around neap tides were more likely to get flooded (average 18%, 95% CI: 2 to 35%) than nests initiated during spring tides (average 12%, 95% CI: 0 to 29%; Fig. 5b, Table 1).

### Temporal variation in nest initiation

Overall, we found that the number of initiated nests declined over the laying season (Figs. 4 and 5c, Table 2). Consistent with our fourth prediction, parents initiated more nests around spring tides (average 1.03 nests per day, 95% CI: 0.7 to 1.35) than around neap tides (average 0.88 nests per day, 95% CI 0.55 to 1.21; Fig. 5d, Table 2). Accordingly, parents initiated more nests when tides were high as is the case during spring tides. Contrary to our fifth prediction, there was no statistically clear change in nest initiation pattern over the semi-lunar cycle across the laying season (Table 2, ‘complex models’).

## Discussion

Adaptation to tidal rhythms is commonly found in organisms living in intertidal zones [12, 49]. Consistent with our three predictions about temporal variation in nest flooding, we found that the nesting ecology of ground-nesting Snowy Plovers followed variation in tide height at three different timescales: (1) nest flooding varied between years (Fig. 3), (2) was more likely later in the season (Figs. 4 and 5a), and (3) followed the semi-lunar cycle with nests initiated during neap tides more likely to be flooded than nests initiated during spring tides (Fig. 5b). Consistent with adaptation to the semi-lunar tide changes, more nests were initiated around the time of spring tides than around neap tides (Fig. 5d). By contrast, our last prediction was not supported as the strength of the semi-lunar nest initiation rhythm did not change clearly over the season (Table 2). Below we discuss these findings in detail.

### Temporal variation in nest flooding

The observed high between-year stochasticity in nest flooding is consistent with findings from other avian species [19, 20, 32, 37] and likely reflects the between-year differences in extreme weather events when spring tides coincide with onshore gales that push the waterline further inland than regular weather. As previously suggested [19, 20, 37], the stochasticity of these weather events makes adaptation to changing water levels particularly challenging. Despite these nest losses caused by stochastic flooding events, plovers seemed well adapted to breed in intertidal habitat as only 6% of nests failed due to flooding (Fig. 4).

We observed a strong seasonal effect on the risk of nest flooding. Nearly all flooding events affected nests laid during the second part of the laying season (Fig. 4). One potential reason for this is that tide height increases over the breeding season and the nest distribution of the plovers shifts towards areas that are more susceptible to tidal flooding. Thus, late breeding plovers might trade off nest survival with chick survival. As the high evaporation in late spring leads to a retreat of the water bodies, the chick survival of late hatching nests is greatly reduced [14] because the chicks do not find enough food on the dry salt flats and often starve to death unless the nest is initiated close to shoreline where the flooding risk is higher. Another reason for the seasonal trend in nest flooding is that tropical storms are more likely to occur late in the season at this site (CK, MCL, SGA personal observations).

Finally, the flooding risk was associated with the semi-lunar rhythm. Nests initiated around spring tides were less likely to be flooded than nests initiated during neap tides. The average flooding risk for nests initiated during neap tide was on average 50% higher than for nests initiated during spring tide although the uncertainty of the estimates was high and likely caused by only 6% of nests being flooded in this population.

### Temporal variation in nest initiation

Consistent with an adaptive response to the semi-lunar variation in nest flooding, Snowy Plovers at Bahía de Ceuta initiated more nests around the dates of spring tides than during the dates of neap tides (Fig. 5d). Nest initiation before and after spring tide may be adaptive for different reasons. For example, nest initiation at or shortly after a spring tide will enable the parents to pick a flood-safe nesting spot. In contrast, initiation just before spring tide will ensure that chicks hatch right at the spring tide and have the shortest distance to feeding territories as the nesting period is slightly longer than two semi-lunar cycles. Parents may use at least two cues associated with semi-lunar periodicity to initiate nests at a favourable time in order to reduce the risk of nest flooding. Similar to other marine species [24, 50, 51], parents may use periodic changes in moonlight levels to anticipate the occurrence of spring tides and time their nesting activity accordingly. This would require the regulation of reproduction in female plovers, for example, through an endogenous circa-lunar clock [24]. To date, such clocks have not been described in higher vertebrates [12]. Alternatively, plovers could track the actual high tide levels and use the associated water line directly to inform themselves about safe nesting sites.

Given that high spring tides and the start of the rain season will terminate the laying season in early July, late nesting plovers might be pressed for time and could be forced to deviate from adaptive semi-lunar timing, so the semi-lunar periodicity of nest initiation should get weaker over the season. Contrary to our prediction and despite our large sample of more than 700 nests the interaction between spring tide cycle number and nest initiation rates during the semi-lunar cycle was weak and not clearly supported in our models. However, we note that the observed weak trend was consistent with our prediction, i.e. nest initiation rates seemed to show a stronger adherence to the semi-lunar cycle and tide height at the beginning of the season whereas there was no such a relationship at the end of the season (Table 2).

Temporal adaptation in response to nest flooding has been previously dismissed as unlikely mechanism in some temperate coastal birds [19]. Indeed, nest flooding through tides, despite being the second most important reason for nest failure after predation [14], was responsible for only a small proportion of nest failures in our population. Moreover, despite data on 752 nests collected over 10 years, the semi-lunar periodicity of nest initiation in our Snowy Plover population was only moderate. There might be methodological and biological reasons for such a weak relationship, and we discuss six in detail. First, we did not measure tide height at the site (or at each nest) but instead relied on predicted tide heights for a station located about 95 km away. Second, for the majority of nests, the true nest initiation date was estimated based on categorical egg floating charts assuming a generic rate for the egg laying period. Third, plovers might choose more flood-safe nest locations especially when they initiate nests at lower tides. However, choosing nest locations that are safe from flooding can compromise nest survival in other ways [17, 20], e.g. by providing inferior conditions for the hatchlings leading to increased chick mortality [14, 22] or via increased risk of nest predation [52–56]. Predation risk may therefore be traded off with the risk of flooding, as flood-safe nests that are further away from the shoreline or more elevated might be more vulnerable to predation [21, 23]. Indeed, Snowy Plovers nesting on artificial nest mounds were more likely to survive extreme weather events, yet it is unclear whether such nests were also more prone to predation [32]. Fourth, phenotypic plasticity may enable inhabitants of tidal wetlands to adapt to nest flooding but previous studies suggested that not all species are able to do so. For example, Oystercatchers *Haematopus ostralegus* chose seemingly more elevated nest sites after experiencing nest flooding but when tested formally it turned out that their nest site selection was not different from random [20]. In contrast, female Saltmarsh Sparrows *Ammospiza caudacutus* that experienced nest flooding built their replacement clutches more elevated in vegetation than expected by chance [21]. Fifth, parents may be able to reduce the exposure of their nests to spring tides by shortening the incubation period. This can be achieved by increasing the nest temperature, for example, by higher nest attendance [57, 58]. Higher ambient temperatures later in the breeding season may also contribute to shorter incubation times, e.g. as seen in late nests of Snowy Plovers in California [27], as these late nests then experience more favourable thermal conditions than early nests when unattended. Too high ambient temperatures can eventually become a threat for the developing embryos although parents can mitigate this threat by increasing nest attendance and shading the eggs [34, 59]. A shorter nest period by approximately three days would help to avoid the exposure of the immobile clutch to a second spring tide if the nests are initiated right after spring tide. Finally, further cues and pressures might be more important drivers of nest initiation than the time within the spring tide cycle. For example, females may align their reproductive physiology and nest initiation to the availability of partners [60, 61], use certain social cues such as the sight of successful broods [62, 63] or the nesting activity of conspecific or heterospecific females such as Least Tern *Sternulla antillarum* that nest nearby [36, 64].

## Conclusions

We conclude that the temporal patterns of nest initiation and flooding observed in Snowy Plovers are consistent with adaptation required for successful breeding in intertidal habitats. We note that similar to other coastal birds the nesting season coincides with the lowest spring tides of the annual cycle [19], and overall only a small proportion of nests were flooded. The associations of nest initiation rates and flooding with semi-lunar tidal cycles were moderate, and detection required large sample sizes suggesting that there are other adaptive mechanisms contributing to reduce nest failure due to tidal flooding. Yet, an adaptive mechanism relying on timing in regard to the spring tides should be less costly than choosing nesting sites that are more elevated (hence more exposed to predators) and/or further away from preferred feeding habitat. Plovers seem well adapted to the threat of nest flooding that occurs in tidal wetlands. Understanding how birds adapt to nest flooding is essential, especially given that global environmental changes are predicted to increase volatility of climate and the occurrence of extreme climatic events, leading to more flooding [37, 65]. Regular occurring cyclones are important for Snowy Plovers since they create the preferred nesting habitat [66]. Yet, fewer but more extreme storms and sea-level rise are predicted to increase the vulnerability of coastal populations because elevated storm tides will increase nest flooding [19], and further reduce the available habitat for intertidal specialists such as Snowy Plovers [66, 67]. We call for further studies on the significance of timing of nest initiation, preferably to test the generality of adaptive nest initiation in response to spring tides in coastal birds.

## Abbreviations

CI: Credible interval
GPS: Global Positioning System receiver
